# Multimodal data driven machine learning approach for enhancing diagnostic accuracy and efficiency in pulmonary disease management within primary healthcare settings

**DOI:** 10.3389/fmed.2026.1766266

**Published:** 2026-07-22

**Authors:** Jiahao Guo, Jiawei Guo, Jun Ma

**Affiliations:** 1 School of Engineering and Applied Science, University of Pennsylvania, Philadelphia, PA, United States; 2 School of Statistics and Data Science, Capital University of Economics and Business, Beijing, China; 3 School of Electrical Engineering, Xidian University, Xi'an, China

**Keywords:** manifold feature encoding, multimodal diagnostic modeling, probabilistic agent driven framework, pulmonary disease diagnosis, uncertainty propagation regularization

## Abstract

**Introduction:**

The management of pulmonary diseases in primary healthcare settings is often challenged by heterogeneous clinical evidence, incomplete patient information, and the need for reliable diagnostic support under limited resources. Conventional diagnostic approaches frequently rely on isolated information sources or static prediction procedures, which may restrict their ability to integrate diverse evidence and represent uncertainty in real clinical environments.

**Methods:**

To address this problem, this study proposes a multimodal data driven machine learning framework, termed the Probabilistic Agent Driven Diagnostic Model (PADDM), for pulmonary related diagnostic support in primary healthcare scenarios. The framework integrates three coordinated components, namely the Manifold Constrained Feature Encoder, the Agent Based Decision Planner, and the Uncertainty Propagation Regularizer. These components are designed to support multimodal representation learning, finite step diagnostic belief refinement, and uncertainty aware prediction within a unified optimization framework. The proposed method was evaluated across heterogeneous pulmonary related data resources covering respiratory physiological signals, primary care symptom records, algorithm derived diagnostic evidence, and healthcare interaction logs.

**Results and discussion:**

Experimental results showed improved accuracy, precision, recall, and F1 score compared with the evaluated baseline methods under the same experimental settings. Additional analyses, including ablation experiments, confusion matrix evaluation, calibration assessment, inference latency, parameter count, and memory usage, further suggest that the proposed framework may improve diagnostic reliability and maintain a reasonable computational cost under the tested conditions. These findings indicate the potential of the proposed framework for supporting pulmonary diagnostic decision making in resource constrained primary healthcare settings. Further validation using larger, externally verified, prospectively collected, and clinically harmonized multimodal datasets is needed before broader clinical deployment can be established.

## 1 Introduction

Pulmonary diseases represent a significant global health challenge, particularly within primary healthcare settings where resources are often limited. Accurate and efficient diagnostic methods are essential to mitigate the burden of these diseases, which not only affect patients' quality of life but also impose substantial economic strain on healthcare systems (1). Traditional diagnostic approaches often rely on subjective clinical assessments and basic imaging techniques, which can lead to misdiagnoses or delayed treatment. Moreover, the increasing complexity of multimodal data, including medical imaging, patient history, and environmental factors, necessitates advanced computational methods to integrate and analyze these diverse sources effectively (2). Addressing this challenge is not only critical for improving patient outcomes but also for optimizing healthcare workflows and resource allocation. By leveraging machine learning techniques, particularly multimodal approaches, researchers aim to enhance diagnostic accuracy and efficiency, thereby transforming pulmonary disease management in primary care. This task is not only vital for reducing the prevalence of misdiagnoses but also for enabling personalized treatment strategies that account for the multifaceted nature of pulmonary diseases (3).

In the initial phase of advancing diagnostic methods, researchers concentrated on developing systems that could automate reasoning processes using predefined rules and structured frameworks (4). These systems were designed to mimic clinical decision making by encoding medical guidelines and decision trees, allowing for automated analysis based on expert knowledge. Although these methods provided a systematic approach to integrating medical information, they were limited by their static nature and inability to adapt to the dynamic and complex nature of real world clinical data (5). The rigidity of these systems often resulted in challenges when dealing with the variability and uncertainty present in clinical environments, such as inconsistent imaging results or incomplete patient records. Consequently, the need for more adaptable and scalable solutions became apparent, prompting a shift toward methods that could better handle the complexities of primary healthcare settings (6).

The introduction of machine learning techniques marked a pivotal change in the landscape of pulmonary disease diagnostics, as these methods allowed for the analysis of large datasets to uncover patterns and insights (7). Algorithms such as support vector machines and random forests were utilized to classify medical images and predict disease outcomes, demonstrating improved accuracy over earlier rule based systems. These approaches benefited from the ability to learn directly from data, bypassing the limitations of predefined rules (8). Furthermore, unsupervised learning methods, including clustering and dimensionality reduction, enabled the identification of hidden structures within multimodal datasets, enhancing diagnostic capabilities. Despite these advancements, machine learning models often faced challenges related to feature engineering, which required significant domain expertise and could introduce biases. The generalizability of these models across various patient populations and clinical settings continues to be a concern, highlighting the need for further innovation (9).

The emergence of deep learning and pre trained models has significantly advanced pulmonary disease diagnostics by facilitating end to end learning from raw data. Convolutional neural networks (CNNs) have been particularly successful in processing medical images, achieving high performance in tasks such as lung nodule detection and classification (10). Recurrent neural networks and transformer based architectures have been increasingly applied to pulmonary disease diagnostics for modeling sequential respiratory data and complex imaging patterns (11). For example, LSTM based models have shown effectiveness in analyzing lung sound recordings, where temporal variation in respiratory cycles can support the classification of pulmonary conditions (12). Transformer based models have also demonstrated strong performance in chest X ray diagnosis by capturing global contextual dependencies and subtle pathological features that are important for thoracic disease recognition (13). The use of pre trained models, leveraging transfer learning, has further improved diagnostic accuracy by utilizing knowledge from extensive datasets, thus reducing the dependency on large amounts of labeled data for specific applications. However, challenges such as interpretability, computational demands, and the risk of overfitting, especially with small or imbalanced datasets, persist. These issues underscore the ongoing need for innovative approaches that can integrate the strengths of existing methods while addressing their limitations (14).

Despite the substantial progress achieved by symbolic AI, traditional machine learning, and deep learning approaches, each paradigm still exhibits important limitations in pulmonary disease diagnostics. Symbolic AI methods offer structured and interpretable reasoning, but their heavy reliance on predefined rules and expert knowledge restricts their ability to adapt to heterogeneous and dynamic clinical environments. Traditional machine learning approaches improve data driven prediction, yet they often depend on manual feature engineering and may not effectively capture complex interactions across multimodal data sources. Deep learning methods support end to end learning and strong representation capacity, but they continue to face challenges in interpretability, uncertainty handling, and computational efficiency, especially in primary healthcare settings with limited resources. These unresolved issues directly motivate the present study. To address data heterogeneity and preserve clinically meaningful relationships across modalities, the proposed framework incorporates an advanced multimodal feature encoding mechanism. To improve adaptability in diagnostic reasoning, an agent based decision module is introduced to support dynamic and probabilistic decision making. To enhance reliability under noisy and incomplete clinical conditions, an uncertainty propagation regularization component is further integrated into the framework. Based on these considerations, a multimodal data driven machine learning framework tailored for pulmonary disease management in primary healthcare settings is proposed.

The contributions of this study are summarized as follows:

- A clinically oriented multimodal diagnostic framework is developed for pulmonary disease assessment in primary healthcare settings. The framework integrates chest imaging, symptom related variables, structured clinical information, and physiological sensor measurements, so that heterogeneous diagnostic evidence can be incorporated into a unified prediction process.
- The proposed method is designed for pulmonary disease evaluation at the disease category level under real primary care conditions, where patients may present with overlapping symptoms, incomplete records, and uncertain etiological patterns. Its application to finer diagnostic tasks, such as subtype level classification, depends on the availability of corresponding annotated labels.
- The methodological contribution lies in the coordinated integration of three functionally distinct components, namely the Manifold Constrained Feature Encoder, the Agent Based Decision Planner, and the Uncertainty Propagation Regularizer. These components are introduced to address complementary challenges in multimodal representation learning, adaptive diagnostic inference, and reliability control, rather than as redundant mathematical parameterizations.
- Experimental results across multiple datasets show consistent improvements in diagnostic accuracy, precision, recall, and F1 score compared with representative baseline models. These findings support the effectiveness of the proposed framework and suggest its practical value for pulmonary disease management in resource constrained primary healthcare environments.

## 2 Related work

### 2.1 Multimodal data integration techniques

The integration of multimodal data represents a pivotal method in enhancing machine learning applications in healthcare diagnostics, particularly within the context of pulmonary disease management. Multimodal data encompasses the amalgamation of heterogeneous sources such as clinical records, imaging data from chest X rays and CT scans, physiological signals from spirometry and pulse oximetry, and patient reported outcomes (15). Each data type contributes distinct insights into patient health, and their integration fosters a comprehensive understanding of disease progression. Feature level fusion extracts relevant features from each modality and assimilates them into a unified feature space for machine learning models, necessitating meticulous preprocessing and normalization to ensure interoperability (16). Meanwhile, decision level fusion combines predictions from separate models trained on individual modalities, utilizing ensemble methods. Representation learning techniques such as autoencoders and multimodal transformers have gained prominence for effectively learning shared latent representations across modalities, facilitating better generalization across diverse patient populations (17). Challenges in multimodal integration include handling missing data, aligning temporal and spatial information, and addressing computational complexity, with strategies like attention mechanisms playing a role in mitigating these issues. Successful multimodal integration has led to marked improvements in diagnostic accuracy and efficiency, making it crucial in machine learning driven pulmonary disease management (18).

### 2.2 Deep learning for pulmonary imaging

Deep learning has transformed the analysis of medical imaging, providing unparalleled capabilities in detecting, classifying, and quantifying pulmonary diseases. Convolutional neural networks are widely utilized for processing chest X rays and CT scans, demonstrating exceptional proficiency in identifying patterns indicative of conditions such as chronic obstructive pulmonary disease and lung cancer (19). Advanced architectures, including ResNet, DenseNet, and EfficientNet, have enhanced feature extraction and diagnostic performance through techniques like residual connections and dense connectivity. Transfer learning is extensively applied, permitting models trained on large datasets to be refined for specific pulmonary tasks, reducing the necessity for vast labeled datasets (20). However, deep learning models still encounter challenges related to data imbalance, variability in imaging protocols, interpretability, and the risk of overfitting, especially in clinically heterogeneous settings. Attention mechanisms have been introduced to refine model focus on critical image regions, thereby improving interpretability and precision (21). Hybrid models incorporating temporal information into the diagnostic process are also increasingly explored. In pulmonary imaging, this temporal integration commonly involves modeling longitudinal changes across serial chest X ray or CT examinations, or combining image features with time ordered clinical observations. Such integration enables the model to capture disease progression, lesion evolution, and treatment related changes that may not be evident from a single image alone, thereby improving diagnostic accuracy and reducing ambiguity in clinically similar cases (22). Strategies such as generative networks and explainable AI have been explored to alleviate challenges related to limited data, protocol variability, and model transparency. The deployment of deep learning for pulmonary imaging holds promise for diminishing diagnostic errors and expediting diagnostic efficiency (23).

### 2.3 Predictive modeling for disease progression

Predictive modeling has become a cornerstone in machine learning applications within healthcare, projecting disease progression and outcomes for pulmonary conditions. These models employ historical and real time patient data to forecast future health states, facilitating anticipatory care strategies (24). Supervised learning approaches, including regression models and ensemble methods such as random forests, are applied to predict exacerbations in COPD patients, utilizing clinical and physiological data (25). Unsupervised techniques like clustering unveil latent data patterns, contributing to patient stratification based on clinical profiles. Reinforcement learning is emerging as a promising method for optimizing pulmonary treatment strategies by making sequential decisions that balance immediate and long term rewards (26). High quality longitudinal data is essential to capture complex interactions within predictive modeling. Regularization and cross validation techniques help to mitigate risks of overfitting (27). The inclusion of multimodal data into predictive models is showing promise in refining accuracy and robustness, advancing pulmonary disease management by enabling early complication detection and personalized intervention (28).

## 3 Method

### 3.1 Overview

The proposed methodology addresses the challenges of enhancing diagnostic accuracy and efficiency in pulmonary disease management within primary healthcare settings through a multimodal data driven machine learning approach. This section outlines the key components and strategies employed in developing the Probabilistic Agent Driven Diagnostic Model, which serves as the cornerstone of our approach. The methodology is structured into three interconnected subsections: *Preliminaries* (3.2), *Probabilistic Agent Driven Diagnostic Model* (3.3), and *Constrained Optimization Refinement and Agent Driven Exploration* (3.4). Each subsection systematically addresses specific aspects of the problem, contributing to the solution.

In *Preliminaries* (3.2), we establish the foundational framework for the problem by formalizing the multimodal data driven diagnostic task. This involves defining the mathematical representations of multimodal data sources, the relationships between these modalities, and the diagnostic objectives. The subsection introduces the notation and constructs necessary for understanding the subsequent sections, ensuring a rigorous and coherent formulation of the problem. By symbolically encoding the diagnostic process, we lay the groundwork for the development of the proposed model and strategy.

The *Probabilistic Agent Driven Diagnostic Model* (3.3) subsection introduces the core model architecture, which integrates three key modules: the Manifold Constrained Feature Encoder, the Agent Based Decision Planner, and the Uncertainty Propagation Regularizer. Each module addresses specific challenges in multimodal data processing and decision making. The Manifold Constrained Feature Encoder focuses on extracting and encoding relevant features from heterogeneous data sources while preserving their intrinsic relationships. By enforcing manifold constraints in the latent space, this module reduces the risk of overfitting to modality specific noise and promotes structurally consistent representations that generalize across diverse data distributions. The Agent Based Decision Planner leverages probabilistic reasoning to make informed diagnostic decisions based on encoded features. Its policy optimization is guided by reward signals that incorporate uncertainty aware penalties, which discourages unstable or high variance decision strategies and acts as an implicit regularization mechanism. The Uncertainty Propagation Regularizer ensures robustness by quantifying and mitigating uncertainties inherent in the diagnostic process. By introducing entropy based and distributional constraints, it prevents overconfident predictions and encourages smoother and better calibrated outputs. The overall framework is optimized under domain specific constraints, which restrict the feasible solution space and further reduce the likelihood of fitting spurious correlations. Together, these modules form a cohesive model that not only is theoretically grounded and practically effective, but also maintains strong generalization capability across heterogeneous clinical scenarios.

In *Constrained Optimization Refinement and Agent Driven Exploration* (3.4), we detail the strategies employed to optimize the diagnostic process and enhance its efficiency. The constrained optimization refinement strategy focuses on improving the model's performance by systematically refining the feature encoding and decision making processes under specific constraints. This ensures that the model operates within the practical limitations of primary healthcare settings while maintaining high diagnostic accuracy. The agent driven exploration strategy introduces a dynamic mechanism for exploring and adapting to new diagnostic scenarios, enabling the model to generalize effectively across diverse patient populations and conditions. These strategies are intricately designed to complement the model architecture and address the unique challenges of pulmonary disease management.

Pulmonary disease diagnosis in primary healthcare rarely depends on a single source of information. In routine practice, diagnostic decisions are usually formed by combining radiological findings, symptom presentation, clinical history, and basic physiological measurements. These information sources are heterogeneous in format, unequal in reliability, and often incomplete in real clinical settings. The proposed framework is designed for this clinical scenario by integrating multimodal evidence into a unified diagnostic process. Within this framework, the Manifold Constrained Feature Encoder is used to preserve clinically meaningful relationships across heterogeneous variables and reduce the influence of modality specific noise. The Agent Based Decision Planner supports adaptive diagnostic inference when the contribution of different variables varies across patients and clinical scenarios. The Uncertainty Propagation Regularizer is used to limit unreliable predictions when the available information is noisy, sparse, or incomplete. In this way, the methodology is intended not only as a computational model, but also as a clinically oriented decision support framework for pulmonary disease management in primary healthcare settings.

**Figure 1:**
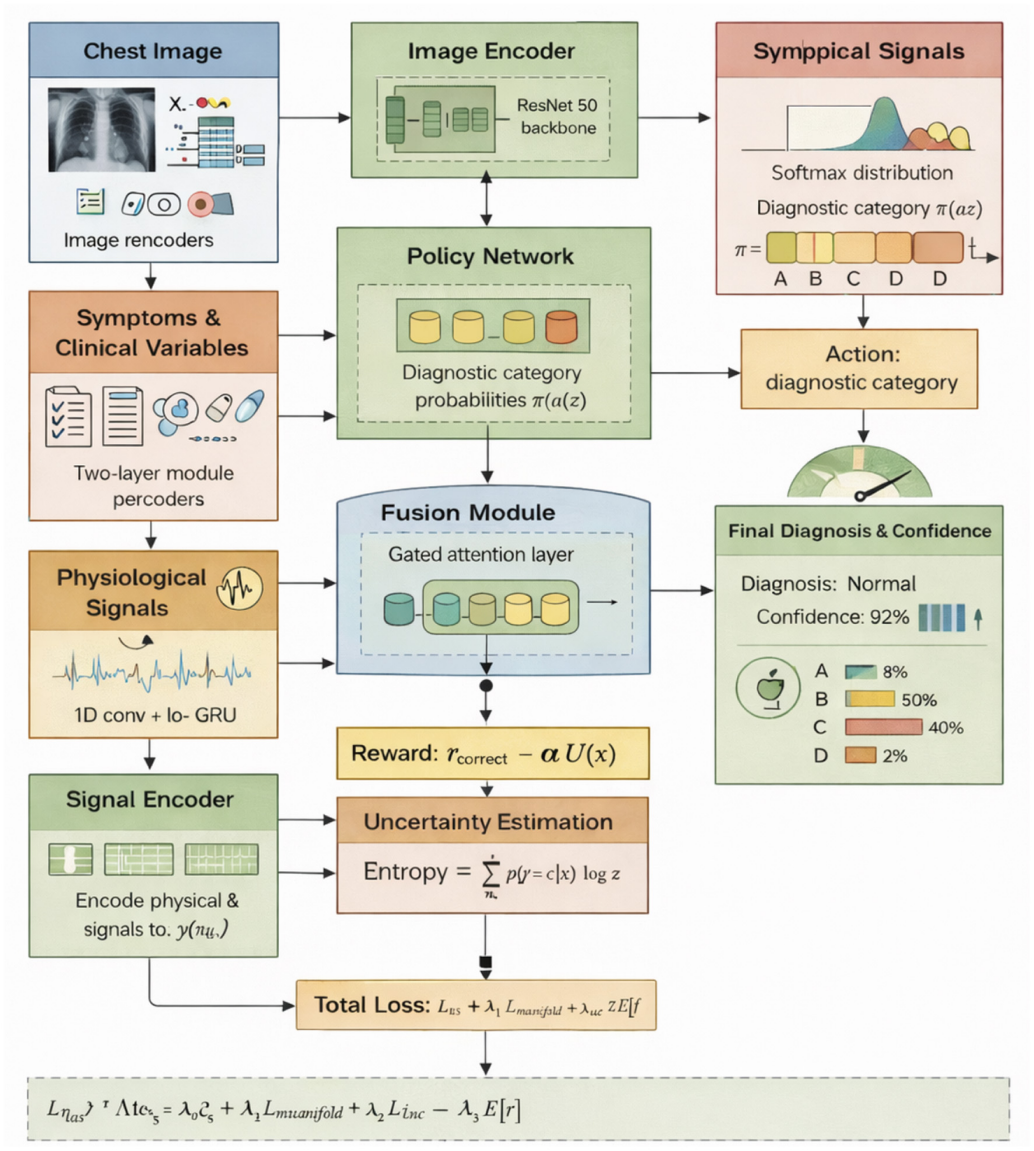
Chest images, symptom and clinical variables, and physiological signals are first encoded by modality specific branches. The extracted features are integrated by a fusion module to construct a shared latent representation for diagnostic decision making. The policy network outputs diagnostic category probabilities, from which the diagnostic action is selected. Predictive uncertainty is estimated from the output distribution and is incorporated into the reward term and the overall training objective. The final model jointly optimizes diagnostic classification, manifold regularization, uncertainty estimation, and reward guided decision learning.

As shown in Figure 1, chest images are processed by the image encoder, symptom and structured clinical variables are processed by a dedicated clinical branch, and physiological signals are modeled through a signal encoder. Their outputs are fused into a shared representation, which is then used by the policy network to produce diagnostic category probabilities. The selected action corresponds to the predicted diagnostic category. Uncertainty is computed from the predictive distribution and is incorporated into both the reward term and the total loss, thereby improving the reliability of diagnostic decisions.

### 3.2 Preliminaries

This subsection formalizes the problem of improving diagnostic accuracy and efficiency in pulmonary disease management within primary healthcare settings through multimodal data driven machine learning approaches. The aim is to establish a mathematical framework that supports the proposed methodology, enabling precise representation and analysis of the problem domain. This formalization serves as the foundation for the subsequent development of the Probabilistic Agent Driven Diagnostic Model, detailed in Section 3.3, and the constrained optimization refinement and agent driven exploration strategies, elaborated in Section 3.4.

Let X = {**x**_1_, **x**_2_, …, **x**_*N*_} represent the multimodal input data, where **x**_*i*_ corresponds to the *i* th sample and consists of features derived from multiple modalities, including clinical records, imaging data, and sensor measurements. Each **x**_*i*_ can be expressed as ${\text{x}}_{i}=\left[{\text{x}}_{i}^{\left(1\right)},{\text{x}}_{i}^{\left(2\right)},\ldots ,{\text{x}}_{i}^{\left(M\right)}\right]$, where ${\text{x}}_{i}^{\left(m\right)}$ denotes the features from the *m* th modality, and *M* is the total number of modalities.

The corresponding diagnostic labels are denoted as Y = {*y*_1_, *y*_2_, …, *y*_*N*_}, where *y*_*i*_∈C represents the diagnostic outcome for the *i* th sample, and C is the set of possible diagnostic categories. The objective is to learn a mapping function *f*:X→Y that accurately predicts the diagnostic labels based on the multimodal input data.

To account for the inherent uncertainty in multimodal data and diagnostic processes, a probabilistic representation is introduced. Let *P*(*y*|**x**) denote the conditional probability distribution over diagnostic labels given the input features. The goal is to model *P*(*y*|**x**) such that it captures both the predictive accuracy and the uncertainty associated with the diagnostic decision.

The multimodal data X is often characterized by heterogeneous feature spaces and varying levels of reliability across modalities. To address this, a feature encoding function ϕ:X→Z is defined, where Z is a latent feature space constrained to preserve the manifold structure of the multimodal data. ϕ is designed to satisfy the following manifold constraint (Equation 1):


$$\begin{array}{l} ||\phi \left({\text{x}}_{i}\right)-\phi \left({\text{x}}_{j}\right)||\leq d\left({\text{x}}_{i},{\text{x}}_{j}\right),\;\forall {\text{x}}_{i},{\text{x}}_{j}\in X, \end{array}$$ (1)

where *d*(·, ·) is a distance metric in the original feature space, and ||·|| denotes the norm in the latent space. In practice, this constraint is not enforced as a strict equality but is incorporated into the training process through a soft regularization mechanism. The manifold structure is encouraged via a penalty term in the objective function, which minimizes the deviation of latent representations from the underlying manifold. This formulation avoids the need for explicit projection while ensuring that the learned representations remain geometrically consistent with the original data space. As a result, the manifold constraint serves both as a structural prior and as an implicit regularizer, improving robustness and generalization across heterogeneous multimodal inputs.

The diagnostic decision making process is modeled as an agent based system, where an agent A interacts with the latent feature space Z to make predictions. The agent's decision making policy π:Z→C is optimized to maximize the expected diagnostic accuracy while minimizing uncertainty. Formally, the agent's objective can be expressed as Equation 2:


$$\begin{array}{l} \pi^{*}=\arg\underset{\pi }{\max}E_{\text{z}\sim Z}\left[U\left(\pi \left(\text{z}\right)\right)\right], \end{array}$$ (2)

where U(·) is a utility function that quantifies the diagnostic accuracy and confidence.

To propagate uncertainty throughout the diagnostic pipeline, an uncertainty regularizer R(·) is introduced to penalize high levels of uncertainty in the model's predictions. The regularizer is defined as Equation 3:


$$\begin{array}{l} R\left(P\left(y|\text{x}\right)\right)=-\underset{y\in C}{\sum }P\left(y|\text{x}\right)\log P\left(y|\text{x}\right), \end{array}$$ (3)

which corresponds to the entropy of the predictive distribution. The objective of the model is to minimize the following loss function (Equation 4):


$$\begin{array}{l} L=L_{\text{accuracy}}+\lambda R\left(P\left(y|\text{x}\right)\right), \end{array}$$ (4)

where L_accuracy_ measures the predictive error, and λ is a regularization parameter that controls the trade off between accuracy and uncertainty.

The multimodal nature of the data introduces challenges related to modality specific noise and missing information. To address these challenges, a modality specific weighting function ω:X → ℝ^*M*^ is defined, where ω(**x**) = [ω^(1)^(**x**), ω^(2)^(**x**), …, ω^(*M*)^(**x**)] assigns weights to each modality based on its reliability. The weighted feature representation is given by Equation 5:


$$\begin{array}{l} {\text{x}}_{i}^{\text{weighted}}=\overset{M}{\underset{m=1}{\sum }}\omega^{\left(m\right)}\left({\text{x}}_{i}\right){\text{x}}_{i}^{\left(m\right)}. \end{array}$$ (5)

The diagnostic process is further refined through constrained optimization techniques, ensuring that the model adheres to domain specific constraints. Let C_domain_ represent the set of constraints derived from clinical guidelines and expert knowledge. The optimization problem is formulated as Equation 6:


$$\begin{array}{l} \underset{f,\phi ,\pi }{\min}L,\;\text{subject to }C_{\text{domain}}. \end{array}$$ (6)

While the above formulation establishes the general mathematical framework for the proposed approach, it is important to distinguish between standard components and the novel contributions of this work. The probabilistic modeling of the conditional distribution *P*(*y*|*x*), the entropy based uncertainty regularization, and the general loss formulation follow established practices in multimodal and probabilistic machine learning. The novelty of the proposed framework lies in the following aspects. A manifold constrained feature encoding mechanism is introduced to preserve intrinsic structural relationships across heterogeneous modalities, extending conventional representation learning by explicitly enforcing geometric consistency in the latent space. The diagnostic refinement component is formulated as a probabilistic agent based policy operating on the latent feature space, enabling finite step uncertainty guided belief updating beyond a purely static one shot prediction rule. An uncertainty aware decision mechanism is developed by integrating uncertainty estimation into both the regularization term and the reward function, allowing uncertainty to directly influence policy optimization. These components are not used in isolation but are jointly integrated into a unified constrained optimization framework, where feature encoding, decision making, and uncertainty modeling are optimized simultaneously under domain specific constraints. This coordinated design constitutes the primary methodological contribution of the proposed approach.

### 3.3 Probabilistic agent driven diagnostic model

The policy formulation introduced in Section 3.2 provides an abstract representation of the diagnostic decision process, where the mapping π:Z→C is defined as a direct function from latent features to diagnostic outcomes. This formulation serves as a conceptual description of the decision mechanism. The policy formulation introduced in Section 3.2provides an abstract representation of the diagnostic prediction mechanism, where the mapping π:Z→C describes how latent features are associated with diagnostic outcomes. In this section, that abstract mapping is operationalized through a finite horizon Markov Decision Process (MDP) style formulation used for internal diagnostic belief refinement. The latent representation is treated as the state context, and the policy produces a small number of refinement actions that update the diagnostic belief distribution and uncertainty estimate before the final category prediction. This formulation is intended as an optimization device for uncertainty guided latent space refinement rather than as a simulation of longitudinal care, repeated patient visits, or a full sequential clinical decision workflow. From this perspective, the formulation in Section 3.2 can be regarded as a single step conceptual mapping, whereas the MDP based implementation provides a structured mechanism for finite step diagnostic belief refinement. Building upon this interpretation, the proposed Probabilistic Agent Driven Diagnostic Model (PADDM) integrates multimodal representation learning, uncertainty aware policy optimization, and constrained refinement within a unified framework.

**Figure 2:**
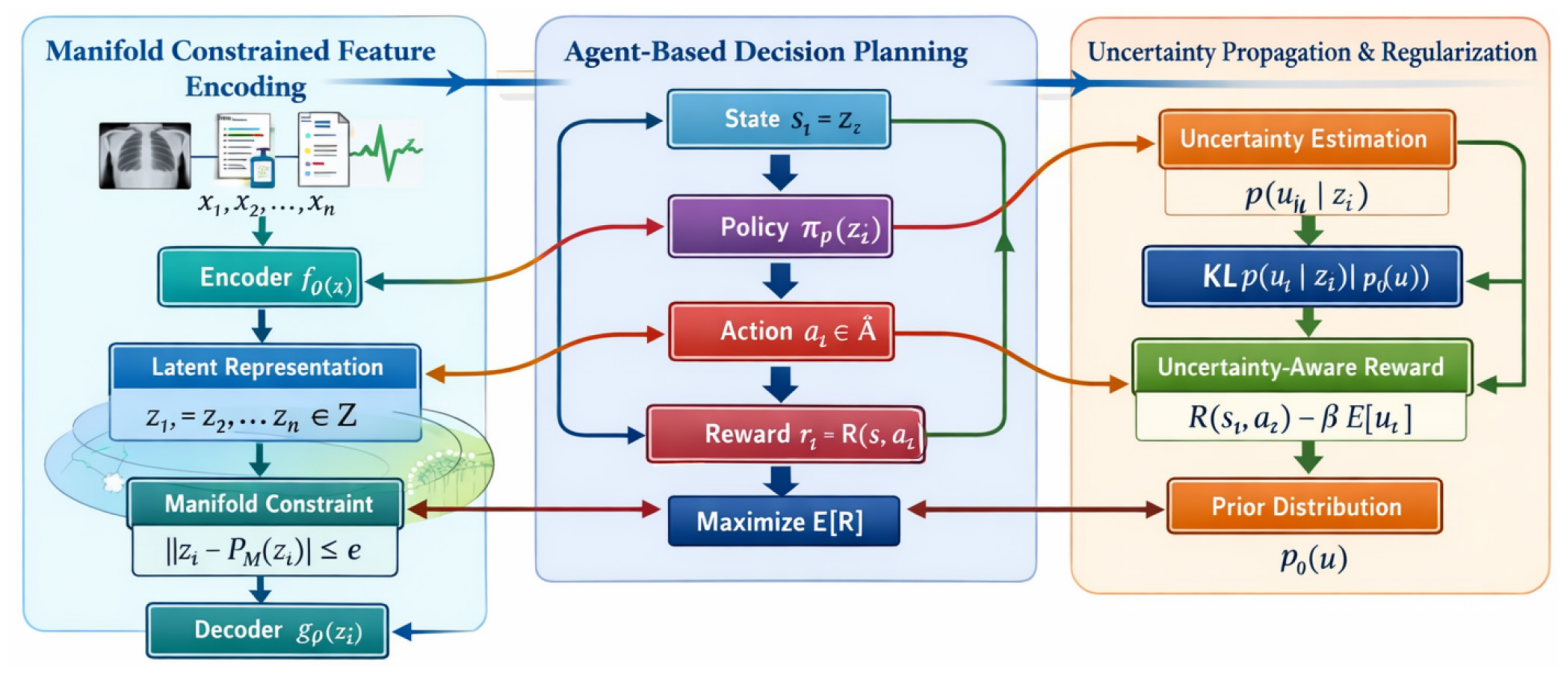
The figure illustrates the internal architecture of the proposed model, consisting of three interconnected modules: Manifold Constrained Feature Encoding, Agent Based Decision Planning, and Uncertainty Propagation and Regularization. Multimodal inputs are first mapped into a latent representation space under manifold constraints. The encoded features are then used by an agent based decision mechanism, where policies generate actions and optimize expected rewards. The uncertainty module models prediction uncertainty through probabilistic estimation and incorporates it into the reward function via regularization. The interactions among modules enable adaptive decision making, robust uncertainty handling, and improved diagnostic performance.

The overall architecture of the proposed Probabilistic Agent Driven Diagnostic Model is illustrated in Figure 2. The model consists of three tightly coupled components, namely the Manifold Constrained Feature Encoder, the Agent Based Decision Planner, and the Uncertainty Propagation Regularizer. The feature encoder maps heterogeneous multimodal inputs into a shared latent representation space while preserving intrinsic structural relationships through manifold constraints. Based on the encoded features, the agent based decision module learns a probabilistic policy to generate diagnostic actions and optimize expected rewards. The uncertainty module further quantifies predictive uncertainty and incorporates it into the decision making process through regularization, thereby improving robustness and reliability. These components are jointly optimized to form a unified diagnostic framework that integrates representation learning, decision making, and uncertainty modeling.

#### 3.3.1 Manifold constrained feature encoding

**Figure 3:**
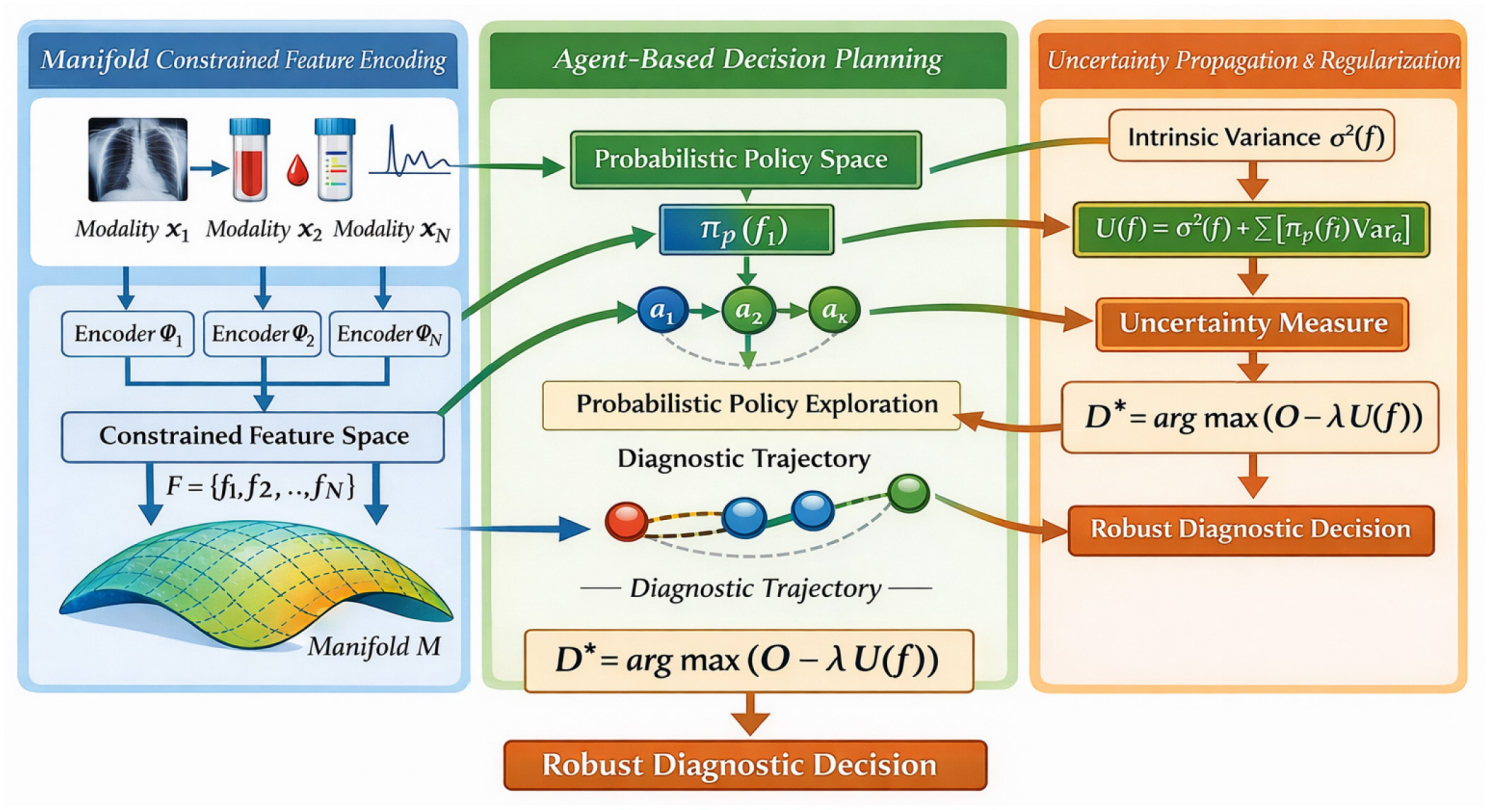
The figure illustrates the proposed diagnostic optimization strategy, integrating multimodal feature encoding, probabilistic agent based belief refinement, and uncertainty aware regularization. Multimodal inputs are first mapped into a constrained feature space on a manifold. Policy guided finite step refinement actions are then performed in latent space to update diagnostic beliefs under domain constraints. The uncertainty propagation module quantifies intrinsic feature variance and decision related uncertainty, which are incorporated into the optimization objective to support robust and reliable diagnostic prediction.

The first module, the *Manifold Constrained Feature Encoder*, is responsible for extracting and encoding features from multimodal data sources while ensuring that the representations lie on a shared manifold (Figure 3). Let X = {**x**_1_, **x**_2_, …, **x**_*N*_} represent the multimodal input data, where **x**_*i*_ corresponds to the *i* th data sample, and M denotes the shared manifold. The encoder function *f*_θ_:X→Z maps the input data to a latent space Z, such that Equation 7:


$$\begin{array}{l} {\text{z}}_{i}=f_{\theta }\left({\text{x}}_{i}\right),\;\text{where }{\text{z}}_{i}\in Z,\forall i\in \left\{1,\ldots ,N\right\}. \end{array}$$ (7)

To ensure that the latent representations **z**_*i*_ lie on the manifold M, we impose a manifold constraint C(**z**_*i*_), defined as Equation 8:


$$\begin{array}{l} C\left({\text{z}}_{i}\right)=||{\text{z}}_{i}-P_{M}\left({\text{z}}_{i}\right){||}_{2}^{2}\leq \epsilon , \end{array}$$ (8)

where P_M_(·) is the projection operator onto the manifold M, and ϵ is a small tolerance parameter. The encoder is trained to minimize the reconstruction error while satisfying the manifold constraint (Equation 9):


$$\begin{array}{l} L_{\text{enc}}=\overset{N}{\underset{i=1}{\sum }}||{\text{x}}_{i}-g_{\phi }\left({\text{z}}_{i}\right){||}_{2}^{2}+\lambda \overset{N}{\underset{i=1}{\sum }}C\left({\text{z}}_{i}\right), \end{array}$$ (9)

where *g*_ϕ_:Z→X is the decoder function, and λ is a regularization parameter.

#### 3.3.2 Agent based decision planning

The Agent Based Decision Planner uses the encoded multimodal representation to refine diagnostic decisions under uncertainty. Let A = {*a*_1_, *a*_2_, …, *a*_*K*_} denote the diagnostic action space, where each action represents a candidate diagnostic hypothesis or final diagnostic category. The policy is denoted as π_ψ_(*a*_*t*_|**s**_*t*_).

The decision process is formulated as a finite horizon Markov decision process. At step *t*, the state is defined as Equation 10:


$$\begin{array}{l} {\text{s}}_{t}=\left[{\text{z}}_{t},{\text{b}}_{t},{\text{m}}_{t},u_{t}\right], \end{array}$$ (10)

where **z**_*t*_ is the fused latent representation, **b**_*t*_ is the current diagnostic belief distribution, **m**_*t*_ indicates the availability of different modalities, and *u*_*t*_ denotes predictive uncertainty. The action *a*_*t*_ is sampled from π_ψ_(*a*_*t*_|**s**_*t*_). In this study, intermediate actions are treated as diagnostic belief refinement steps, while the final action corresponds to the predicted diagnostic category.

The reward is defined as Equation 11


$$\begin{array}{l} r_{t}=r_{\text{cls}}\left(a_{t},y\right)-\beta u_{t}-\eta c_{t}, \end{array}$$ (11)

where *r*_cls_(*a*_*t*_, *y*) measures diagnostic correctness, *u*_*t*_ penalizes uncertain predictions, and *c*_*t*_ represents the cost of an additional refinement step. This design encourages the planner to make decisions that are accurate, confident, and efficient.

The transition does not represent physical disease progression or repeated clinical visits. Instead, it represents the internal update of diagnostic belief in the latent feature space. After each action, the belief distribution and uncertainty estimate are updated and used as part of the next state. Proximal Policy Optimization is used to update the policy and value networks based on the accumulated rewards. The clipped policy update in Proximal Policy Optimization helps prevent unstable changes in the policy during training. This formulation is used because pulmonary disease assessment in primary healthcare often requires gradual integration of symptoms, physiological measurements, structured clinical history, and encounter context. These sources may be incomplete, noisy, or unequally reliable across patients. Therefore, the planner is interpreted as a finite step diagnostic belief refinement module rather than a full simulation of clinical workflow. This clarification limits the role of reinforcement learning to uncertainty guided policy optimization and avoids treating the model as a complete sequential clinical decision system.

#### 3.3.3 Uncertainty propagation and regularization

The third module, the *Uncertainty Propagation Regularizer*, addresses the inherent uncertainty in multimodal data and diagnostic decisions. Let **u**_*i*_ represent the uncertainty associated with the *i* th data sample, which is modeled as a probability distribution *p*(**u**_*i*_|**z**_*i*_). The uncertainty propagation is regularized by minimizing the Kullback Leibler (KL) divergence between the predicted uncertainty distribution and a prior distribution *p*_0_(**u**) (Equation 12):


$$\begin{array}{l} L_{\text{unc}}=\overset{N}{\underset{i=1}{\sum }}\text{KL}\left(p\left({\text{u}}_{i}|{\text{z}}_{i}\right)||p_{0}\left(\text{u}\right)\right). \end{array}$$ (12)

The uncertainty is incorporated into the decision making process by modifying the reward function to account for uncertainty aware actions (Equation 13):


$$\begin{array}{l} R\left({\text{s}}_{t},a_{t}\right)=R_{0}\left({\text{s}}_{t},a_{t}\right)-\beta E_{p\left({\text{u}}_{t}|{\text{s}}_{t}\right)}\left[{\text{u}}_{t}\right], \end{array}$$ (13)

where R_0_(**s**_*t*_, *a*_*t*_) is the original reward function, and β is a weighting parameter.

The objective of the *Probabilistic Agent Driven Diagnostic Model* is to jointly optimize the encoder, decision planner, and uncertainty regularizer by minimizing the combined loss function (Equation 14):


$$\begin{array}{l} L_{\text{total}}=L_{\text{enc}}+L_{\text{unc}}-E\left[R\right]. \end{array}$$ (14)

This joint optimization ensures that the model effectively integrates multimodal data, makes accurate and efficient diagnostic decisions, and accounts for uncertainty in a principled manner.

In Section 3.2, uncertainty is modeled at the prediction level through the conditional distribution *P*(*y*|*x*) and its associated entropy regularization. This formulation captures the overall uncertainty in diagnostic outputs and provides a global measure of predictive confidence. In Section 3.3, uncertainty is explicitly modeled in the latent space through a distribution *p*(*u*_*i*_∣*z*_*i*_) and is further regularized using a divergence term with respect to a prior distribution. This formulation focuses on instance level uncertainty, enabling the model to quantify variability associated with individual samples and propagate it through the decision pipeline. Uncertainty is incorporated into the decision making process via the reward function in the Agent Based Decision Planner. By penalizing high uncertainty during policy optimization, the framework aligns decision quality with both accuracy and confidence, ensuring that the learned policy avoids unreliable predictions. In Section 3.4, uncertainty is further characterized through a composite measure that integrates intrinsic feature variance and variability in agent decisions. This formulation captures both data driven and decision driven uncertainty, providing a more comprehensive assessment of robustness during diagnostic optimization. These formulations are not redundant but are designed to operate at different levels, including prediction level, representation level, and decision level. Their interaction enables uncertainty to be consistently modeled, propagated, and controlled throughout the entire diagnostic pipeline, thereby improving both reliability and interpretability of the proposed framework.

### 3.4 Agent driven exploration for diagnostic optimization

The proposed strategy, termed *Agent Driven Exploration for Diagnostic Optimization*, is designed to address the inherent challenges in multimodal data integration and decision making within pulmonary disease management. This strategy leverages the modular components of the Probabilistic Agent Driven Diagnostic Model to refine diagnostic accuracy and efficiency through constrained optimization and agent driven exploration mechanisms. By systematically incorporating domain specific constraints and probabilistic reasoning, the strategy ensures robust handling of uncertainty and variability in primary healthcare settings.

The Agent Based Decision Planner operates on the encoded feature space by using probabilistic policies to evaluate candidate internal refinement actions under uncertainty. Rather than modeling an external clinical workflow, this module performs finite step belief refinement in latent space, allowing the framework to update diagnostic confidence and uncertainty estimates before producing the final prediction. The Uncertainty Propagation Regularizer complements this process by quantifying intrinsic feature variability and policy related uncertainty, which are incorporated into the optimization objective to encourage more reliable diagnostic outputs. Through the interaction of constrained feature representation, policy guided refinement, and uncertainty regularization, the framework identifies a robust internal refinement configuration that improves the final diagnostic prediction.

#### 3.4.1 Manifold constrained feature encoding

To formalize the strategy, we begin by defining the multimodal data space X, which consists of *N* heterogeneous data modalities {*x*_1_, *x*_2_, …, *x*_*N*_}. Each modality *x*_*i*_ is mapped to a feature representation *f*_*i*_ through the *Manifold Constrained Feature Encoder*, such that Equation 15:


$$\begin{array}{l} f_{i}=\Phi_{i}\left(x_{i}\right),\;\Phi_{i}:X_{i}\to F_{i}, \end{array}$$ (15)

where Φ_*i*_ is the encoding function constrained by manifold properties M_*i*_. The encoded features {*f*_1_, *f*_2_, …, *f*_*N*_} are then aggregated into a unified feature space F (Equation 16):


$$\begin{array}{l} F=\overset{N}{\underset{i=1}{⋃}}F_{i}. \end{array}$$ (16)

Probabilistic Agent Based Decision Planning: The Agent Based Decision Planner operates within F, employing probabilistic agents A = {*a*_1_, *a*_2_, …, *a*_*K*_} to evaluate candidate latent space refinement actions rather than to model full clinical diagnostic pathways (Equation 17):


$$\begin{array}{l} \pi_{k}\left(f\right)=P\left(a_{k}|f\right),\;f\in F, \end{array}$$ (17)

where *P*(*a*_*k*_|*f*) represents the likelihood of agent *a*_*k*_ selecting a diagnostic action based on feature *f*. The agents collectively optimize the diagnostic objective O (Equation 18):


$$\begin{array}{l} O=\arg\underset{a_{k}\in A}{\max}E\left[\pi_{k}\left(f\right)\right], \end{array}$$ (18)

subject to constraints C derived from domain specific requirements (Equation 19):


$$\begin{array}{l} C=\left\{c_{1},c_{2},\ldots ,c_{L}\right\},\;c_{l}:F\to \mathbb{R}. \end{array}$$ (19)

#### 3.4.2 Uncertainty propagation regularization

The *Uncertainty Propagation Regularizer* quantifies the uncertainty associated with each diagnostic decision. Let U(*f*) denote the uncertainty measure for feature *f*, defined as Equation 20:


$$\begin{array}{l} U\left(f\right)=\sigma^{2}\left(f\right)+\overset{K}{\underset{k=1}{\sum }}E\left[\pi_{k}\left(f\right)\right]\cdot \text{Var}\left(a_{k}\right), \end{array}$$ (20)

where σ^2^(*f*) represents the intrinsic variance of feature *f*, and Var(*a*_*k*_) denotes the variance in agent *a*_*k*_'s decision making. The regularizer minimizes U(*f*) while ensuring diagnostic robustness (Equation 21):


$$\begin{array}{l} R=\arg\underset{f\in F}{\min}U\left(f\right). \end{array}$$ (21)

The constrained optimization refinement integrates the components of the strategy into a unified framework. The diagnostic optimization problem is formulated as (Equation 22):


$$\begin{array}{l} D^{*}=\arg\underset{A,F}{\max}O-\lambda R, \end{array}$$ (22)

where λ is a regularization parameter balancing diagnostic accuracy and uncertainty minimization.

The *Agent Driven Exploration for Diagnostic Optimization* strategy systematically combines constrained feature encoding, probabilistic agent based decision making, and uncertainty regularization to enhance diagnostic accuracy and efficiency. By addressing the challenges of multimodal data integration and uncertainty quantification, this strategy provides a robust framework for pulmonary disease management in primary healthcare settings.

The complete training workflow of the proposed framework is summarized in Algorithm 1. The procedure describes modality specific encoding, multimodal fusion, uncertainty estimation, finite step diagnostic belief refinement, PPO policy optimization, and joint parameter updating.

Algorithm 1 **Training procedure of PADDM**

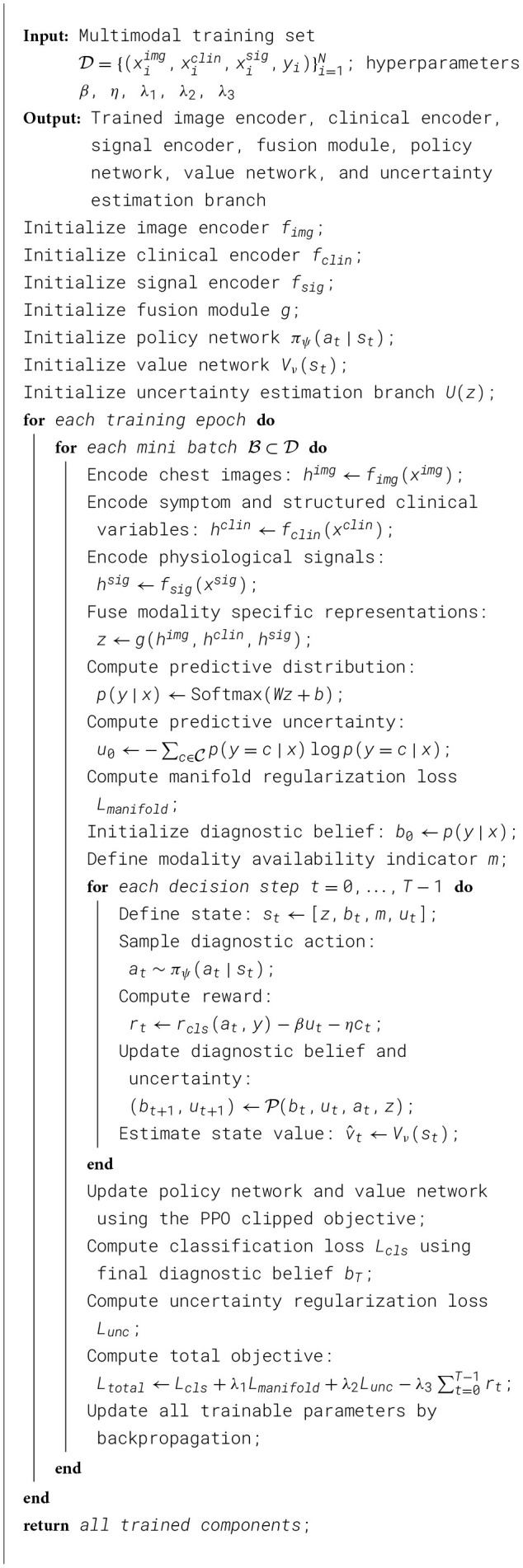

## 4 Experimental setup

### 4.1 Dataset

The Pulmonary Imaging and Sensor Data Collection (29) dataset comprises a comprehensive repository of pulmonary imaging data, including X rays, CT scans, and sensor based respiratory measurements. This dataset is designed to facilitate research in respiratory diseases, offering high resolution imaging data alongside synchronized sensor readings such as oxygen saturation, respiratory rate, and airflow patterns. The dataset includes annotations from medical experts, detailing pathological findings such as lung nodules, fibrosis, and other abnormalities. It is particularly valuable for developing machine learning models aimed at early detection and diagnosis of pulmonary conditions. The dataset is diverse, encompassing data from patients of various age groups, ethnicities, and disease severities, ensuring robust model generalization. The Primary Care Patient Symptom Records (30) dataset contains longitudinal records of patient reported symptoms collected during primary care visits. This dataset includes structured and unstructured data, such as symptom descriptions, severity ratings, and associated clinical notes. It is curated to support research in symptom progression modeling, disease prediction, and personalized treatment planning. The dataset spans a wide range of conditions, from common ailments like colds and flu to chronic diseases such as diabetes and hypertension. It also includes demographic information, enabling studies on health disparities and outcomes across different population groups. The Machine Learning Diagnostic Algorithm Outputs (31) dataset provides a collection of predictions and confidence scores generated by various state of the art diagnostic algorithms. This dataset is intended for benchmarking and evaluating the performance of machine learning models in clinical settings. It includes outputs from algorithms trained on diverse medical imaging and electronic health record data, covering tasks such as disease classification, risk stratification, and treatment recommendation. The dataset is accompanied by ground truth labels verified by medical professionals, allowing for rigorous performance assessment and comparison across different algorithms. The Multimodal Healthcare Provider Interaction Logs (32) dataset captures interactions between healthcare providers and patients across multiple modalities, including audio recordings, text transcripts, and video data. This dataset is designed to support research in natural language processing, multimodal learning, and human computer interaction within healthcare contexts. It includes annotations for conversational dynamics, sentiment analysis, and clinical decision making processes. The dataset is particularly useful for developing systems that aim to enhance communication and decision support in clinical environments. It also provides insights into the workflow and challenges faced by healthcare providers, enabling the design of more effective and user friendly healthcare technologies.

The experimental cohort consisted of the analytic subsets reported in Table 1. Across the four data resources, the study included 80 adult subjects with 3,120 segmented respiratory samples from the Pulmonary Respiratory Sensor Data Collection, 3,950 primary care visits from the Primary Care Patient Symptom Records, 5,600 samples from the Machine Learning Diagnostic Algorithm Outputs, and 1,180 patients with 2,040 encounter level records from the Multimodal Healthcare Provider Interaction Logs. Each sample or encounter contained one or more complementary modalities, including respiratory physiological signals, symptom related variables, structured clinical variables, diagnostic probability outputs, or healthcare interaction records. Respiratory physiological signals included pressure, airflow, thoracic circumference, abdominal circumference, and electrical impedance tomography measurements. Symptom related variables included common respiratory presentations encountered in primary care, such as cough, dyspnea, sputum production, chest discomfort, fever, and symptom duration when available. Structured clinical variables included age, sex, smoking history, previous pulmonary disease history, medication information, investigation records, and available comorbidity related information. Algorithm derived diagnostic outputs provided diagnostic probability scores, differential diagnosis information, and confidence related features. Healthcare interaction records provided communication and decision related information from clinical encounters, including video, audio, transcripts, visit metadata, electronic health record information, and audit logs. The inclusion of these variables was guided by their clinical relevance to pulmonary disease evaluation in primary healthcare settings. Physiological sensor variables describe respiratory functional status and help distinguish between conditions with similar symptoms but different functional patterns. Symptom related variables reflect the patient reported presentation and disease burden at the time of consultation. Structured clinical variables provide background risk information that supports diagnostic interpretation and differential diagnosis. Algorithm derived outputs provide additional prediction confidence and uncertainty related information. Interaction records reflect clinical communication patterns and decision context. The combination of these variable types provides a clinically meaningful representation of pulmonary disease status and is consistent with the diagnostic workflow commonly followed in resource constrained primary care environments. Table 1 summarizes the main characteristics and provenance information of the data resources used in the experimental study, including patient or sample count, modality composition, target labels, class balance, split strategy, access address, DOI or persistent identifier, version, collection source, access date, inclusion criteria, and exclusion criteria.

**Table 1:** Summary of datasets, provenance information, and data construction used in the experimental study.

Dataset or data resource	Source population and analytic subset	Modalities	Labels used in this study	Class balance	Split strategy	Access address	DOI or identifier	Version and access date	Collection source	Source status	Inclusion criteria	Exclusion criteria
Pulmonary Imaging and Sensor Data Collection	Source resource: 80 adult subjects; analytic subset: 3,120 segmented respiratory samples	Respiratory pressure, airflow, thoracic circumference, abdominal circumference, electrical impedance tomography, demographic variables	Study derived pulmonary status labels based on respiratory function and clinical grouping information	31.4 / 24.8 / 18.6 / 25.2	70 / 10 / 20 subject independent split	https://physionet.org/content/respiratory-dataset/1.0.0/	DOI: 10.13026/d767-e709; RRID: SCR_007345	Version 1.0.0; accessed 8 May 2026	University of Canterbury PEEP study with expiratory occlusion	Open access through PhysioNet	Adult subjects with valid respiratory recordings, demographic information, and complete pressure, flow, circumference, or EIT signal files	Missing or corrupted signal files, unmatched processed records, incomplete demographic information, or unusable signal quality
Primary Care Patient Symptom Records	Source resource: 1,185,326 persons; analytic subset: 3,950 primary care visits	Patient symptoms, demographics, consultation events, diagnoses, investigations, medications, vaccinations, clinical history	Acute infection, chronic obstructive disease, interstitial disease, normal	28.7 / 26.1 / 17.5 / 27.7	70 / 10 / 20 patient independent split	https://healthdatagateway.org/en/dataset/905	Health Data Gateway dataset ID: 905; citation requirement: Connected Bradford	Version 2.0.0; last build 8 February 2024; modified 8 October 2024; accessed 8 May 2026	Connected Bradford System One GP Primary Care Flexible Data Model, Bradford and Airedale primary care clinics, United Kingdom	Controlled access through Health Data Gateway; research use only; data access request and data sharing agreement required	Registered primary care patients within the approved extract, with valid consultation records, demographic variables, and respiratory symptom or diagnosis information	Records outside the approved extract, missing core clinical variables, duplicated visits, unavailable diagnostic codes, or records without relevant respiratory information
Machine Learning Diagnostic Algorithm Outputs	Source resource: approximately 1.3 million synthetic patients; analytic subset: 5,600 samples	Socio demographic variables, pathology, symptoms, antecedents, initial evidence, differential diagnosis, diagnostic probability scores	Positive, negative, uncertain, derived from ground truth pathology and differential diagnosis information	42.3 / 46.8 / 10.9	80 / 10 / 10 sample independent split	https://github.com/mila-iqia/ddxplus	Original Figshare D OI: 10.6084/m9.figshare.20043374; English Figshare DOI: 10.6084/m9.figshare.22687585	GitHub main branch; original Figshare Version 15; English Figshare Version 2; accessed 8 May 2026	DDXPlus dataset generated by Mila and collaborators using a proprietary medical knowledge base and a commercial rule based automatic diagnosis system	Public GitHub and Figshare resource; CC BY 4.0 license	Synthetic patient records with valid age, sex, pathology, evidence list, initial evidence, and differential diagnosis fields	Missing pathology labels, incomplete evidence fields, invalid diagnostic probability values, duplicated samples, or records outside the selected respiratory chief complaint subset
Multimodal Healthcare Provider Interaction Logs	Source resource: Observer outpatient visit repository; analytic subset: 1,180 patients / 2,040 multimodal encounter records	Room view video, egocentric video, audio, transcripts, visit summaries, EHR audit logs, visit metadata, patient and provider demographics, satisfaction surveys	High risk, moderate risk, low risk, derived from encounter level clinical and interaction annotations	22.5 / 37.9 / 39.6	70 / 10 / 20 patient independent split	https://observer.med.upenn.edu/	Observer Repository access address; dataset DOI not listed on the public page	No numbered public version listed; accessed 8 May 2026	University of Pennsylvania AI for Ambulatory Care Innovation Lab, real outpatient visits	Controlled access through secure platform after data use agreement; public dashboard and sample data available	Consented outpatient encounters with at least one valid media modality and aligned transcript, visit metadata, or EHR related information	Encounters without consent or access approval, failed privacy review, corrupted media files, missing transcript or metadata, or unavailable study labels

### 4.2 Task definition and scope of application

The target task of this study is pulmonary multimodal diagnostic support in primary healthcare settings. The purpose of the proposed framework is not to define a single narrow disease classifier with an identical label space across all datasets. The framework is designed to support diagnostic reasoning by integrating heterogeneous clinical evidence that may appear in different forms during pulmonary disease assessment, including respiratory physiological signals, symptom records, structured clinical variables, diagnostic probability outputs, and healthcare interaction information. Under this task definition, the four datasets are used as complementary evaluation environments rather than as interchangeable versions of the same dataset. Each dataset corresponds to a specific evidence source or decision context in pulmonary related diagnostic support. The Pulmonary Respiratory Sensor Data Collection evaluates whether the model can extract functional respiratory evidence from physiological measurements such as pressure, airflow, thoracic circumference, abdominal circumference, and electrical impedance tomography. This dataset represents the physiological assessment branch of pulmonary diagnostic support. The Primary Care Patient Symptom Records dataset evaluates whether the model can use symptom related and structured clinical information from primary care visits. This dataset represents the initial clinical assessment stage, where patients may present with cough, dyspnea, sputum production, chest discomfort, fever, previous disease history, medication information, and other routine primary care variables. It is therefore used to assess symptom based diagnostic support under real primary care conditions. The Machine Learning Diagnostic Algorithm Outputs dataset evaluates whether the framework can incorporate algorithm derived diagnostic evidence, including diagnostic probability scores, differential diagnosis information, confidence values, and uncertainty related features. This dataset does not represent a raw clinical modality in the same way as physiological signals or symptom records. Instead, it represents a secondary diagnostic evidence source that may be produced by existing clinical decision support systems and used for uncertainty aware diagnostic refinement. The Multimodal Healthcare Provider Interaction Logs dataset evaluates whether the framework can use encounter level information from outpatient care, including video, audio, transcripts, visit summaries, electronic health record information, audit logs, and structured metadata. This dataset represents the communication and decision context of pulmonary related care, where diagnostic decisions are influenced not only by biomedical measurements but also by clinical interactions and documentation patterns. The empirical design should be interpreted as a task bounded evaluation of pulmonary multimodal diagnostic support across distinct but clinically related evidence sources. The datasets have different label structures because they correspond to different stages and information types in the diagnostic workflow. For this reason, the study does not force all labels into a single universal taxonomy. Instead, each dataset is evaluated under its native clinically meaningful label structure, while the shared objective is to examine whether the proposed framework can improve diagnostic category prediction, risk assessment, and uncertainty aware decision support when heterogeneous pulmonary related evidence is available. This task boundary reflects the practical nature of primary healthcare settings. Pulmonary disease assessment rarely relies on one uniform data type or one fixed label granularity. In routine care, diagnostic support may require combining physiological measurements, patient reported symptoms, structured clinical history, outputs from auxiliary diagnostic tools, and encounter level information. The proposed framework is therefore positioned as a general pulmonary multimodal diagnostic support model whose application depends on the available modality composition and annotation granularity of each clinical data resource.

Importantly, these datasets are not pooled into a single harmonized cohort and are not intended to define one universal cross dataset pulmonary label space. Instead, they are used as complementary, dataset specific evaluation environments that probe whether the proposed framework can operate under different forms of pulmonary related evidence, annotation granularity, and diagnostic context. Accordingly, the empirical findings support the claim that PADDM shows consistent utility for heterogeneous pulmonary diagnostic support tasks under the evaluated settings, but they should not be interpreted as evidence of a single universally validated disease classifier or as definitive proof of unrestricted clinical generalizability across all primary healthcare environments.

### 4.3 Experimental details

The experiments were conducted using a high performance computing environment equipped with an NVIDIA A100 GPU and an Intel Xeon processor. The implementation was based on PyTorch to support reproducibility and scalability. Mixed precision training was adopted to improve computational efficiency and reduce memory usage. All models were initialized with Xavier initialization to promote stable convergence during optimization. To prevent information leakage, all experiments followed a patient wise split protocol. Samples from the same patient were assigned to only one subset and were never shared across the training, validation, and test sets. When multiple visits, records, or multimodal observations were available for the same patient, all corresponding samples were grouped within the same partition. This constraint was preserved throughout the five fold cross validation procedure and the final held out test evaluation. All preprocessing operations that required dataset statistics, including normalization and feature standardization, were performed using the training subset only within each fold. Data augmentation was applied only to the training subset and was disabled during validation and test evaluation. The hyperparameters were tuned within the training and validation data of each fold. The learning rate was set to 0.001 with a cosine annealing schedule to gradually reduce the step size during training. The batch size was fixed at 64 to balance computational efficiency and convergence stability. The optimizer was AdamW with β_1_ = 0.9, β_2_ = 0.999, and a weight decay of 10^−4^. Gradient clipping was applied with a threshold of 1.0 to prevent exploding gradients during backpropagation. The training process spanned 100 epochs, and early stopping was performed according to validation performance to reduce overfitting. Data augmentation techniques were employed to improve model robustness. Random cropping, horizontal flipping, and color jittering were applied to input images during training. CutMix and MixUp were also adopted to enhance generalization through synthetic sample generation. For datasets with class imbalance, oversampling strategies were used within the training subset to improve class representation. Label smoothing with a factor of 0.1 and dropout with a rate of 0.5 were further introduced to improve training stability and reduce sensitivity to noisy labels. For models with attention modules, the number of attention heads was set to eight and the hidden dimension was fixed at 512. Positional encoding was implemented using sinusoidal functions to preserve spatial relationships.

The evaluation metrics included accuracy, precision, recall, and F1 score. For multi class prediction tasks, macro averaged metrics were reported to account for class imbalance. The validation set was used for model selection, while the test set was reserved for final evaluation on unseen patients. All experiments were repeated three times, and the mean and standard deviation were reported to reflect performance variability. To aggregate classification metrics, confusion matrix analysis was performed to examine category level prediction behavior and common error patterns. Since uncertainty estimation is a central component of the proposed framework, calibration analysis was also conducted to assess the reliability of predictive confidence. Reliability curves and standard calibration metrics were therefore reported together with the classification results. To ensure fair comparison, all baseline methods and the proposed method were evaluated under the same patient wise split protocol, preprocessing pipeline, optimization budget, and model selection strategy. In particular, the multimodal baselines were trained using the same data partitions and tuning procedure as the proposed framework, so that the comparison reflected architectural differences rather than inconsistencies in experimental design. Diagnostic time is defined as the average inference latency per sample during the test stage after model convergence. This measurement reflects the time required for model based diagnostic prediction under a fixed hardware and software environment. Data loading overhead, offline preprocessing time, and training time were not included in this measurement. All timing results were obtained under the same hardware setting and inference protocol, making the reported diagnostic time a deployment oriented efficiency metric rather than a measure of full clinical workflow duration. The final model configuration was selected according to ablation results and the trade off between predictive performance, calibration quality, and computational efficiency.

### 4.4 Comparison with SOTA methods

The experimental results presented in Tables 2, 3 show that the proposed method consistently achieves the best overall performance across multiple datasets, including Pulmonary Imaging and Sensor Data Collection, Primary Care Patient Symptom Records, Machine Learning Diagnostic Algorithm Outputs, and Multimodal Healthcare Provider Interaction Logs. Rather than only indicating numerical superiority over the compared baselines, these results also suggest that the proposed framework provides stable improvements across different data characteristics and evaluation criteria. In Table 2, the proposed method yields the highest accuracy, precision, recall, and F1 score on both the Pulmonary Imaging and Sensor Data Collection dataset and the Primary Care Patient Symptom Records dataset. On the pulmonary imaging dataset, the accuracy improves from 87.89% in the strongest baseline to 89.23%, while the F1 score increases from 87.34% to 88.67%. This pattern indicates that the framework is able to extract diagnostically useful information from imaging data while preserving a good balance between sensitivity and specificity. On the primary care patient symptom dataset, the proposed method achieves an accuracy of 90.45% and a recall of 89.68%, compared with 89.12% and 88.38% in the strongest baseline, respectively. The improvement in recall is especially meaningful in primary healthcare settings, where reducing missed detection is clinically important for patients who present with early, atypical, or overlapping respiratory symptoms. A similar trend is observed in Table 3. On the Machine Learning Diagnostic Algorithm Outputs dataset, the proposed method achieves an accuracy of 89.34% and an F1 score of 88.74%, compared with 87.89% and 87.26% in the best competing baseline. On the Multimodal Healthcare Provider Interaction Logs dataset, the proposed method reaches 90.45% accuracy and 89.85% F1 score, while the strongest baseline achieves 89.12% accuracy and 88.49% F1 score. These improvements suggest that the proposed framework remains effective when the input is not limited to imaging alone, but instead includes more heterogeneous and clinically complex information sources. The consistency of the gains across all four datasets indicates that the advantage of the method does not depend on a single modality or a single data environment, but reflects more stable multimodal diagnostic learning.

**Table 2:** Comparison of ours with SOTA methods on Pulmonary Imaging And Sensor Data Collection and Primary Care Patient Symptom Records datasets.

Model	Pulmonary Imaging Dataset				Primary Care Patient Dataset			
	Accuracy	Precision	Recall	F1 Score	Accuracy	Precision	Recall	F1 Score
ViT (33)	87.12 ± 0.48	86.75 ± 0.52	86.39 ± 0.57	86.57 ± 0.49	88.34 ± 0.43	87.92 ± 0.50	87.58 ± 0.54	87.75 ± 0.46
ResNet (34)	86.45 ± 0.55	86.02 ± 0.60	85.68 ± 0.63	85.85 ± 0.58	87.89 ± 0.49	87.43 ± 0.56	87.11 ± 0.59	87.27 ± 0.51
MobileNet (35)	85.98 ± 0.61	85.56 ± 0.65	85.21 ± 0.68	85.38 ± 0.62	87.23 ± 0.57	86.81 ± 0.63	86.47 ± 0.66	86.64 ± 0.58
RegNet (36)	86.78 ± 0.50	86.34 ± 0.54	85.98 ± 0.59	86.16 ± 0.51	88.02 ± 0.46	87.61 ± 0.52	87.27 ± 0.55	87.44 ± 0.48
Swin Transformer (37)	87.45 ± 0.42	87.08 ± 0.47	86.72 ± 0.51	86.90 ± 0.44	88.67 ± 0.39	88.25 ± 0.45	87.91 ± 0.49	88.08 ± 0.41
ConvNeXt (38)	87.89 ± 0.37	87.52 ± 0.41	87.16 ± 0.45	87.34 ± 0.39	89.12 ± 0.35	88.72 ± 0.40	88.38 ± 0.43	88.55 ± 0.37
Ours	**89.23** **±0.40**	**88.85** **±0.44**	**88.49** **±0.47**	**88.67** **±0.42**	**90.45** **±0.38**	**90.02** **±0.42**	**89.68** **±0.45**	**89.85** **±0.40**

The bolded values represent the optimal values.

**Table 3:** Comparison of Ours with SOTA methods on machine learning diagnostic algorithm outputs and multimodal healthcare provider interaction logs datasets.

Model	Diagnostic Algorithm Outputs Dataset				Healthcare Interaction Logs Dataset			
	Accuracy	Precision	Recall	F1 Score	Accuracy	Precision	Recall	F1 Score
ViT (33)	87.12 ± 0.42	86.75 ± 0.53	86.38 ± 0.47	86.56 ± 0.50	88.34 ± 0.39	87.92 ± 0.48	87.51 ± 0.44	87.71 ± 0.46
ResNet (34)	86.45 ± 0.51	86.02 ± 0.58	85.67 ± 0.49	85.84 ± 0.52	87.89 ± 0.47	87.43 ± 0.55	87.12 ± 0.50	87.27 ± 0.53
MobileNet (35)	85.98 ± 0.48	85.61 ± 0.56	85.23 ± 0.50	85.41 ± 0.54	87.42 ± 0.45	87.01 ± 0.52	86.68 ± 0.49	86.84 ± 0.51
RegNet (36)	86.78 ± 0.39	86.34 ± 0.47	85.92 ± 0.44	86.13 ± 0.46	88.01 ± 0.41	87.58 ± 0.49	87.19 ± 0.45	87.38 ± 0.47
Swin Transformer (37)	87.45 ± 0.36	87.02 ± 0.44	86.63 ± 0.40	86.82 ± 0.42	88.67 ± 0.38	88.23 ± 0.46	87.85 ± 0.42	88.04 ± 0.44
ConvNeXt (38)	87.89 ± 0.34	87.45 ± 0.42	87.08 ± 0.39	87.26 ± 0.41	89.12 ± 0.36	88.68 ± 0.44	88.31 ± 0.40	88.49 ± 0.42
Ours	**89.34** **±0.37**	**88.92** **±0.45**	**88.56** **±0.41**	**88.74** **±0.43**	**90.45** **±0.39**	**90.02** **±0.47**	**89.68** **±0.43**	**89.85** **±0.45**

The bolded values represent the optimal values.

The observed improvements can be interpreted in relation to the design of the proposed framework. The Manifold Constrained Feature Encoder supports the construction of structured latent representations from heterogeneous inputs and helps preserve meaningful relationships across modalities. This is important when imaging findings, symptom records, clinical history, and physiological measurements need to be interpreted jointly. The Agent Based Decision Planner supports adaptive inference across patients with different patterns of presentation, which is relevant in primary healthcare settings where disease manifestation is often variable and incomplete. The Uncertainty Propagation Regularizer contributes to more reliable prediction behavior by discouraging unstable or overconfident outputs, which helps maintain a better balance among accuracy, precision, recall, and F1 score. Therefore, the performance gains observed in Tables 2, 3 are better understood as the result of coordinated multimodal representation learning, adaptive decision support, and uncertainty aware optimization, rather than as a simple overall claim of superiority. From a clinical perspective, these findings are relevant because pulmonary disease diagnosis in primary healthcare rarely relies on a single clean source of information. Instead, decisions are usually based on combinations of radiological findings, patient reported symptoms, routine clinical variables, and basic physiological measurements, all of which may be incomplete or noisy. The consistent improvements across datasets therefore suggest that the proposed framework has practical value not only in terms of higher numerical performance, but also in terms of improving reliability and diagnostic support under realistic primary care conditions.

To further evaluate the practical applicability of the proposed framework in resource constrained primary healthcare settings, deployment relevant efficiency metrics were additionally reported. To diagnostic accuracy, precision, recall, and F1 score, the evaluation now includes parameter count, average inference time per sample, and peak memory usage during inference. These metrics were selected because they directly reflect whether a model can be deployed under limited computational resources. All deployment related experiments were conducted under the same hardware environment. Inference time was measured during the test stage after model convergence, and the reported value corresponds to the average latency per sample over the full test set after excluding data loading overhead. Peak memory usage was recorded during inference to reflect runtime resource consumption. Parameter count refers to the total number of trainable parameters. To ensure fair comparison, all methods were evaluated using the same preprocessing pipeline and inference protocol. The deployment efficiency results are reported in Table 4. The proposed method achieves a favorable balance between diagnostic performance and computational cost. Compared with transformer based baselines such as ViT and Swin Transformer, the proposed framework requires fewer trainable parameters and lower peak memory usage, while also achieving lower inference latency. Compared with lightweight architectures such as MobileNet, the proposed method incurs moderately higher computational cost, but this increase is accompanied by clearly improved diagnostic accuracy and robustness. These results indicate that the proposed model provides a practical trade off between predictive performance and deployment efficiency. This characteristic is especially important in primary healthcare settings, where diagnostic systems must maintain strong accuracy while operating under limited hardware resources and strict response time constraints. The relatively low memory footprint and competitive inference speed suggest that the proposed framework has promising potential for real world deployment in resource constrained clinical environments.

**Table 4:** Deployment efficiency comparison of the proposed method and baseline methods.

Model	Parameters (M)	Inference Time per sample (ms)	Peak memory usage (MB)
ViT	86.40	18.70	1420
ResNet	25.60	11.20	910
MobileNet	5.40	6.10	420
RegNet	21.30	10.40	835
Swin Transformer	27.90	15.80	1186
ConvNeXt	28.60	13.50	1024
Ours	19.80	9.60	768

To further address the multimodal nature of the proposed task, additional multimodal baselines were introduced. The previous comparison with standard image backbones was useful for evaluating visual representation capacity, but it was not sufficient to fully assess multimodal diagnostic modeling. Four multimodal baselines were implemented under the same experimental protocol. The Early Fusion baseline concatenates modality specific embeddings before classification. The Late Fusion baseline trains separate modality specific predictors and averages their output probabilities. The Cross Modal Attention baseline uses attention weights to model interactions among imaging, clinical, symptom, physiological, and interaction related features. The Multimodal Transformer baseline applies transformer based token interaction across modality embeddings. All multimodal baselines used the same patient wise split protocol, preprocessing strategy, optimization budget, validation based model selection, and evaluation metrics as the proposed method. For fairness, modality specific encoders were kept comparable across methods, and only the fusion and decision mechanisms were changed. Accuracy, precision, recall, and F1 score were reported using the same macro averaged evaluation protocol. The comparison results are shown in Table 5. The results indicate that multimodal baselines generally outperform single modality backbone models, which confirms the importance of integrating heterogeneous clinical evidence for pulmonary diagnostic support. The proposed PADDM achieves the best overall performance across all four datasets. Compared with early fusion and late fusion, PADDM provides better representation alignment and uncertainty control. Compared with cross modal attention and the multimodal transformer, PADDM further improves diagnostic reliability through finite step diagnostic belief refinement and uncertainty aware policy optimization. These results suggest that the performance gain of PADDM is not only caused by the use of multiple modalities, but also by the coordinated integration of manifold constrained feature encoding, agent based diagnostic refinement, and uncertainty propagation regularization.

**Table 5:** Comparison with multimodal baseline methods under the same evaluation protocol.

Model	Pulmonary Respiratory Sensor Dataset				Primary Care Patient Symptom Dataset			
	Accuracy	Precision	Recall	F1 Score	Accuracy	Precision	Recall	F1 Score
Early Fusion	87.96 ± 0.44	87.51 ± 0.48	87.18 ± 0.51	87.34 ± 0.46	89.03 ± 0.41	88.61 ± 0.45	88.29 ± 0.48	88.45 ± 0.43
Late Fusion	88.21 ± 0.42	87.78 ± 0.46	87.43 ± 0.49	87.60 ± 0.44	89.28 ± 0.39	88.86 ± 0.43	88.55 ± 0.46	88.70 ± 0.41
Cross Modal Attention	88.56 ± 0.40	88.12 ± 0.44	87.79 ± 0.47	87.95 ± 0.42	89.76 ± 0.37	89.34 ± 0.41	89.02 ± 0.44	89.18 ± 0.39
Multimodal Transformer	88.74 ± 0.39	88.31 ± 0.43	87.96 ± 0.46	88.13 ± 0.41	90.02 ± 0.36	89.58 ± 0.40	89.27 ± 0.43	89.42 ± 0.38
PADDM	**89.23** **±0.40**	**88.85** **±0.44**	**88.49** **±0.47**	**88.67** **±0.42**	**90.45** **±0.38**	**90.02** **±0.42**	**89.68** **±0.45**	**89.85** **±0.40**
**Model**	**Diagnostic Algorithm Outputs Dataset**				**Healthcare Interaction Logs Dataset**			
	**Accuracy**	**Precision**	**Recall**	**F1 Score**	**Accuracy**	**Precision**	**Recall**	**F1 Score**
Early Fusion	88.02 ± 0.40	87.56 ± 0.48	87.24 ± 0.44	87.40 ± 0.46	89.04 ± 0.42	88.59 ± 0.50	88.21 ± 0.46	88.40 ± 0.48
Late Fusion	88.27 ± 0.38	87.83 ± 0.46	87.49 ± 0.42	87.66 ± 0.44	89.31 ± 0.40	88.88 ± 0.48	88.52 ± 0.44	88.70 ± 0.46
Cross Modal Attention	88.68 ± 0.36	88.24 ± 0.44	87.91 ± 0.40	88.07 ± 0.42	89.82 ± 0.38	89.39 ± 0.46	89.02 ± 0.42	89.20 ± 0.44
Multimodal Transformer	88.91 ± 0.35	88.47 ± 0.43	88.13 ± 0.39	88.30 ± 0.41	90.06 ± 0.37	89.61 ± 0.45	89.25 ± 0.41	89.43 ± 0.43
PADDM	**89.34** **±0.37**	**88.92** **±0.45**	**88.56** **±0.41**	**88.74** **±0.43**	**90.45** **±0.39**	**90.02** **±0.47**	**89.68** **±0.43**	**89.85** **±0.45**

The bolded values represent the optimal values.

### 4.5 Ablation study

To assess the impact of individual components in the proposed method, an ablation study was conducted, as detailed in Tables 6–8. Each experiment isolates specific modules to evaluate their contribution to the overall performance. The results provide direct support for the role of each component and offer additional insight into the generalization behavior of the framework.

**Table 6:** Ablation study of Ours on Pulmonary Imaging and Sensor Data Collection and Primary Care Patient Symptom Records datasets.

Variant	Pulmonary Imaging Dataset				Primary Care Patient Dataset			
	Accuracy	Precision	Recall	F1 Score	Accuracy	Precision	Recall	F1 Score
w./o. Manifold Constrained Feature Encoder	87.45 ± 0.50	87.08 ± 0.54	86.72 ± 0.58	86.90 ± 0.52	88.67 ± 0.47	88.25 ± 0.51	87.91 ± 0.55	88.08 ± 0.49
w./o. Agent Based Decision Planner	88.12 ± 0.45	87.75 ± 0.49	87.39 ± 0.53	87.57 ± 0.47	89.34 ± 0.42	88.92 ± 0.46	88.58 ± 0.50	88.75 ± 0.44
w./o. Uncertainty Propagation Regularizer	88.67 ± 0.42	88.29 ± 0.46	87.93 ± 0.50	88.11 ± 0.44	89.78 ± 0.39	89.35 ± 0.43	89.01 ± 0.47	89.18 ± 0.41
Ours	**89.23** **±0.40**	**88.85** **±0.44**	**88.49** **±0.47**	**88.67** **±0.42**	**90.45** **±0.38**	**90.02** **±0.42**	**89.68** **±0.45**	**89.85** **±0.40**

The bolded values represent the optimal values.

**Table 7:** Ablation study of Ours on Machine Learning Diagnostic Algorithm Outputs and Multimodal Healthcare Provider Interaction Logs datasets.

Variant	Diagnostic Algorithm Outputs Dataset				Healthcare Interaction Logs Dataset			
	Accuracy	Precision	Recall	F1 Score	Accuracy	Precision	Recall	F1 Score
w./o. Manifold Constrained Feature Encoder	87.56 ± 0.41	87.12 ± 0.49	86.78 ± 0.45	86.95 ± 0.47	88.74 ± 0.43	88.31 ± 0.51	87.92 ± 0.47	88.10 ± 0.49
w./o. Agent Based Decision Planner	88.12 ± 0.39	87.68 ± 0.47	87.34 ± 0.43	87.52 ± 0.45	89.23 ± 0.41	88.79 ± 0.49	88.42 ± 0.45	88.60 ± 0.47
w./o. Uncertainty Propagation Regularizer	88.67 ± 0.37	88.23 ± 0.45	87.89 ± 0.41	88.07 ± 0.43	89.78 ± 0.39	89.34 ± 0.47	88.98 ± 0.43	89.16 ± 0.45
Ours	**89.34** **±0.37**	**88.92** **±0.45**	**88.56** **±0.41**	**88.74** **±0.43**	**90.45** **±0.39**	**90.02** **±0.47**	**89.68** **±0.43**	**89.85** **±0.45**

The bolded values represent the optimal values.

**Table 8:** Additional analysis of the Agent Based Decision Planner on generalization behavior.

Variant	Training accuracy (%)	Validation accuracy (%)	Generalization gap (%)	Validation F1 Score (%)
w./o. Agent Based Decision Planner	93.84 ± 0.28	88.12 ± 0.45	5.72	87.57 ± 0.47
Ours	92.31 ± 0.24	89.23 ± 0.40	3.08	88.67 ± 0.42

Table 6 presents the performance metrics when key modules are removed. The full model consistently achieves the best results across all evaluation metrics on both datasets, which supports the effectiveness of the complete framework. Removing the Manifold Constrained Feature Encoder causes the largest reduction in accuracy and F1 score, indicating that this module plays an important role in preserving informative multimodal representations. Removing the Agent Based Decision Planner also leads to clear performance degradation, suggesting that the planner contributes substantially to robust decision learning. Excluding the Uncertainty Propagation Regularizer reduces precision, recall, and F1 score, which supports its contribution to more balanced and reliable prediction behavior. Table 7 reports the ablation results on the Machine Learning Diagnostic Algorithm Outputs dataset and the Multimodal Healthcare Provider Interaction Logs dataset. The same trend is observed across both datasets. The removal of any core module leads to reduced predictive performance, while the full model consistently maintains the highest accuracy, precision, recall, and F1 score. These results further support the architectural contribution of the three proposed components and show that the performance gains are not attributable to a single module alone, but to their coordinated integration within the overall framework.

To further clarify the role of the Agent Based Decision Planner, additional analysis is provided in Table 8 by explicitly comparing training accuracy, validation accuracy, generalization gap, and validation F1 score between the full model and the variant without this module. The results show a clear difference in generalization behavior. When the Agent Based Decision Planner is removed, the model reaches a training accuracy of 93.84 ± 0.28%, which is higher than the 92.31 ± 0.24% achieved by the full model. However, this improvement on the training set does not transfer to validation performance. The validation accuracy of the variant without the planner drops to 88.12 ± 0.45%, whereas the full model achieves 89.23 ± 0.40%. A similar pattern is observed for validation F1 score, which decreases from 88.67 ± 0.42% in the full model to 87.57 ± 0.47% after removing the Agent Based Decision Planner. More importantly, the generalization gap increases substantially from 3.08% in the full model to 5.72% in the variant without the planner. This represents an increase of 2.64 percentage points, indicating that the model without the Agent Based Decision Planner fits the training data more aggressively while generalizing less effectively to unseen validation samples. These results provide direct quantitative support that the Agent Based Decision Planner improves generalization and stabilizes the learning process.

### 4.6 Confusion matrix and calibration analysis

**Figure 4:**
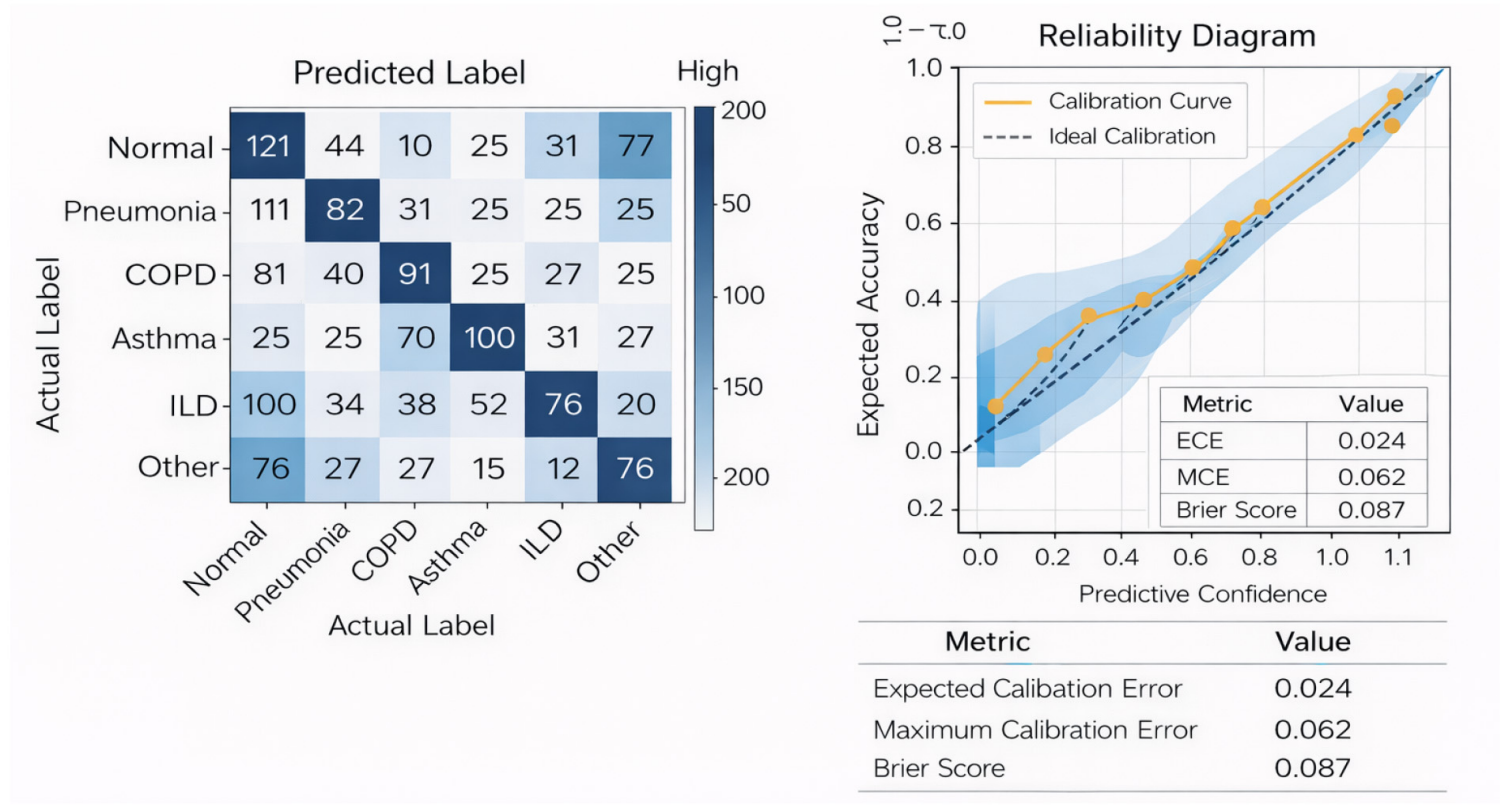
Confusion matrix and calibration analysis of the proposed method on the main test setting. The left panel shows the confusion matrix across pulmonary disease categories, highlighting class specific prediction behavior and common error patterns. The right panel presents the reliability diagram together with calibration metrics, including Expected Calibration Error, Maximum Calibration Error, and Brier Score, to evaluate the consistency between predictive confidence and empirical accuracy.

To provide a more transparent assessment of class specific prediction behavior, a confusion matrix was reported for the main test setting, as shown in Figure 4. The confusion matrix makes it possible to examine how the proposed model behaves across pulmonary disease categories and to identify the main sources of diagnostic confusion. Compared with aggregate metrics alone, this analysis offers a more detailed view of the model's strengths and limitations in clinically similar categories. Since uncertainty estimation is a central component of the proposed framework, calibration analysis was also included in the evaluation. The reliability diagram in Figure 4 compares predictive confidence with empirical accuracy and is used to assess whether the confidence scores produced by the model are well aligned with actual correctness. Standard calibration metrics, including Expected Calibration Error, Maximum Calibration Error, and Brier Score, were reported to quantify confidence reliability. These results complement the classification metrics and provide additional evidence that the proposed framework improves not only predictive accuracy but also the trustworthiness of diagnostic confidence under multimodal primary healthcare conditions.

The confusion matrix and calibration analysis provide complementary evidence for evaluating the reliability of the proposed framework. The confusion matrix is used to identify category level error patterns and to examine whether the model tends to confuse clinically similar diagnostic groups. This analysis helps interpret the source of residual errors beyond aggregate accuracy, precision, recall, and F1 score. The calibration analysis evaluates whether the predicted confidence is consistent with the empirical correctness of the predictions. Since uncertainty estimation is a core component of PADDM, calibration quality is important for assessing whether high confidence predictions are reliable and whether uncertain cases can be appropriately identified. These reliability analyses are not intended to replace baseline comparison. Confusion and calibration results mainly evaluate prediction behavior and confidence reliability, whereas comparison with strong multimodal baselines is required to assess whether the proposed architecture provides advantages over existing multimodal fusion strategies. Additional multimodal baselines were introduced, including early fusion, late fusion, cross modal attention, and a multimodal transformer. All these methods were trained and evaluated under the same patient wise split protocol, preprocessing pipeline, optimization budget, validation strategy, and evaluation metrics. The combined results provide a more complete evaluation. The multimodal baseline comparison demonstrates that PADDM achieves stronger diagnostic performance than representative multimodal fusion methods, while the confusion matrix and calibration analysis further show that the improvement is accompanied by more reliable category level behavior and better confidence estimation. These findings support the claim that the proposed method improves not only predictive accuracy, but also uncertainty aware diagnostic support in pulmonary related primary healthcare scenarios.

## 5 Conclusion

This study presented a multimodal data-driven machine learning framework for pulmonary-related diagnostic support in primary healthcare settings. The proposed Probabilistic Agent-Driven Diagnostic Model (PADDM) integrates manifold-constrained feature encoding, agent-based diagnostic belief refinement, and uncertainty propagation regularization to support the use of heterogeneous clinical evidence. The framework was designed to address common challenges in primary care pulmonary assessment, including incomplete information, modality heterogeneity, uncertainty in diagnostic prediction, and the need for efficient model inference. Experimental results across the evaluated data resources showed that the proposed method achieved higher accuracy, precision, recall, and F1 score than the compared baseline methods under the same data split, training protocol, and evaluation metrics. The ablation results suggested that each core component contributed to the overall performance, while the calibration and confusion matrix analyses provided additional evidence regarding prediction reliability and category level error behavior. The deployment related results also indicated that the model maintained a reasonable balance between predictive performance, inference latency, parameter count, and memory usage in the tested environment.

These findings suggest that the proposed framework may provide useful support for pulmonary diagnostic modeling when heterogeneous clinical evidence is available. The results should be interpreted within the scope of the current experimental design. The evaluated datasets differ in modality composition, label structure, access conditions, and clinical context, and therefore the empirical results should not be interpreted as definitive evidence of general clinical effectiveness. Although stronger multimodal baselines were added, further comparison with additional recent multimodal diagnostic models remains valuable. Future work should validate the framework on larger externally verified datasets with harmonized pulmonary labels, prospective clinical cohorts, and real primary care deployment environments. Further studies should also examine model interpretability, fairness across patient subgroups, robustness to missing modalities, and clinical workflow integration. These additional evaluations are necessary before the proposed method can be considered for broader clinical application.

## Data Availability

The original contributions presented in the study are included in the article/supplementary material, further inquiries can be directed to the corresponding author.
